# Iron deficiency in young basketball players: Is a 100 μg/L ferritin cut‐off appropriate for iron supplementation?: Results of a randomized placebo‐controlled study

**DOI:** 10.1002/clc.24117

**Published:** 2023-09-19

**Authors:** Emese Csulak, Titanilla Takács, Brigitta Babis, Laura Horváth, Petra Márton, Bálint Lakatos, Attila Kovács, Levente Staub, Liliána Erzsébet Szabó, Zsófia Dohy, Hajnalka Vágó, Béla Merkely, Nóra Sydó

**Affiliations:** ^1^ Heart and Vascular Center Semmelweis University Budapest Hungary; ^2^ Department of Internal Medicine and Hematology Semmelweis University Budapest Hungary; ^3^ Honvéd Basketball Academy Budapest Hungary; ^4^ Argus Cognitive, Inc. Lebanon New Hampshire USA; ^5^ Department of Sports Medicine Semmelweis University Budapest Hungary

**Keywords:** athletes, cardiopulmonary exercise test, iron deficiency, sports performance

## Abstract

**Background:**

Iron deficiency (ID) is one of the most common factors that may reduce sports performance, supplementation forms and doses are still not standardized in athletes. Our aim was to assess the iron status of young male basketball players and to study the effect of iron supplementation in a randomized placebo‐controlled study.

**Hypothesis:**

We hypothesized that due to the higher iron demand of athletes, the 100 μg/L ferritin cut‐off may be appropriate to determine the non‐anemic ID.

**Methods:**

During a sports cardiology screening, questionnaires, laboratory tests, electrocardiograms, echocardiography exams, and cardiopulmonary exercise tests were performed. Athletes with ID (ferritin <100 μg/L) were randomized into iron and placebo groups. Ferrous sulfate (containing 100 mg elemental iron [II] and 60 mg ascorbic acid) or placebo (50 mg vitamin C) was administered for 3 months. All exams were repeated after the supplementation period.

**Results:**

We included 65 (age 15.8 ± 1.7 years) basketball players divided into four age groups. Non‐anemic ID was observed in 60 (92%) athletes. After supplementation, ferritin levels were higher in the iron group (75.5 ± 25.9 vs. 54.9 ± 10.4 μg/L, *p* < .01). Ferritin >100 μg/L level was achieved only in 15% of the athletes. There were no differences in performance between the groups (VO_2_ max: 53.6 ± 4.3 vs. 54.4 ± 5.7 mL/kg/min, *p* = .46; peak lactate: 9.1 ± 2.2 vs. 9.1 ± 2.6 mmol/L, *p* = .90).

**Conclusions:**

As a result of the 3‐month iron supplementation, the ferritin levels increased; however, only a small portion of the athletes achieved the target ferritin level, while performance improvement was not detectable.

## Graphical abstract

The effect of 3‐month oral iron supplementation in young, male basketball players—a randomized placebo‐controlled study using the 100 μg/L cut off for iron deficiency.

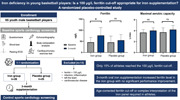

## 1 INTRODUCTION

Iron deficiency (ID) is one of the most common contributing factors that may affect sports performance, although screening, diagnosis, and supplementation have not yet been standardized related to athletes. Compared to the general population, athletes have a higher demand of oxygen transport and energy production, in which iron plays a key role.^1^, ^2^, ^3^ For basketball players, both good cardiovascular endurance and mental concentration are necessary for success. The dynamic part of basketball playing requires a good aerobic capacity. However, several other skills are required to make sudden changes of direction at high speed, such as strength, power, and agility. In this combined (endurance and non‐endurance) team sport, although the significance of an optimal nutritional status is potentially highly important, relevant studies are not yet available.^4^, ^5^

The main causes of ID are the higher iron demand and loss with lower intake and absorption. In athletes, all these four mechanisms are pronounced due to extreme physical burden and the special nutrition habits (intake of supplements, vitamins, and minerals).^3^ Young and/or female athletes are at a higher risk of ID as a result of increased iron requirements due to menstruation, growth, and development.^6^ Therefore, it is essential to diagnose ID in the preclinical stage called iron deficiency without anemia (IDNA) to prevent performance decrement.^7^ ID in female athletes has been studied extensively, though there are only limited data to indicate that IDNA also occurs in young male athletes.^8^, ^9^ There is no guideline for iron supplementation dose, length and frequency,^1^, ^3^, ^10^ nor is there a widely accepted ferritin cut‐off level in athletes, but ferritin >100 μg/L seems to be effective in improving the functional capacity and life quality in specific populations (e.g., heart failure patients).^11^, ^12^, ^13^ A separate decrease in ferritin level indicates IDNA while other iron panel laboratory parameters remain unchanged.^14^ In an oral iron administration, it usually takes at least 3 months to refill the iron storage.^3^, ^15^

The present randomized placebo‐controlled study aims to assess the prevalence of ID and the effect of 3‐month oral iron supplementation in young, male basketball players. We focus on the laboratory parameters, the body composition, and the cardiopulmonary exercise testing (CPET) results before and after the supplementation. We hypothesized that due to the higher iron demand of athletes, the 100 μg/L ferritin cut‐off may be appropriate to determine the IDNA status. On this basis, we investigated whether a 3‐month oral iron supplementation improves laboratory and performance parameters in these young, male athletes with IDNA.

## 2 METHODS

During a detailed sports cardiology screening, all the athletes underwent the following exams: specific sport and nutrition questionnaire, laboratory test, resting electrocardiogram (ECG), body composition analysis, echocardiography, and CPET exam.

### 2.1 Participants

The participants were athletes playing in a Basketball Academy. Our inclusion criteria were the following: (1) male athletes, (2) age under 19 years, (3) no previously known cardiovascular disease. Athletes belonged to U15, U16, U18, and U19 (U = under) age groups. In basketball, “U groups” are categorized according to age and technical abilities. For the randomized placebo‐controlled study, we included athletes with ID defined as ferritin level <100 μg/L. Our exclusion criteria included: (1) clinical or laboratory sign of infection or inflammation; (2) underlying cardiovascular disease detected on the screening exam; (3) athletes, who left the Basketball Academy and could not participate in the control exam.

### 2.2 Sports cardiology screening

Baseline and 3‐month control detailed sports cardiology screening was carried out on all players between September 2020 and June 2021. The athletes were instructed to avoid training for 24 hours before the evaluations and to do their morning routine, including having breakfast and staying hydrated.

Personal and family history with current medical status including medical complaints were assessed on a sport‐specific questionnaire. Family history was considered positive if the parents or grandparents had sudden cardiac death, heart attack, or stroke at a young age (<55 years). The participants also filled in a food frequency questionnaire (FFQ), assessing their dietary iron intake.^16^ The study was supported by a registered dietitian, who also supervised the supplementation process. All the athletes participated in detailed dietary counseling through a series of educational lectures which provided them with information about the iron content of different foods and emphasized the importance of iron supplementation. However, the athletes were advised not to consume additional supplements during our study period.

Standard 12‐lead resting ECG were done with CardioSoft PC (GE Healthcare). We performed a thorough laboratory test for all the athletes. Besides the routine laboratory parameters qualitative and quantitative complete blood count, renal, and liver function, C‐reactive protein, electrolyte panel, creatine kinase, the iron panel were also assessed (ferritin, transferrin, transferrin saturation, soluble transferrin receptor). ID was defined if the ferritin level was lower than 100 μg/L, without any laboratory or clinical sign of infection.

Body composition analysis was performed with InBody 770 based on bioelectric impedance analysis (InBody Co. Ltd). Echocardiography was performed and interpreted on a GE Vingmed ultrasound system by a licensed cardiologist. In accordance with current guidelines a standard protocol containing 2D loops from parasternal, apical, and subxiphoid views was employed.^17^

To evaluate cardiorespiratory fitness a vita maxima incremental test was performed on a treadmill ergometer (GE T‐2100; Healthcare). The athletes subsequently performed the running test at 10 km/h speed with a progressive workload increment rate of 1% every 60 seconds. The athletes were asked to avoid holding the handrail. Gas exchanges were measured using a breath‐by‐breath automated system (Respiratory Ergostik; Geratherm). Lactate levels were measured from fingertip in the pre‐exercise phase and in every 2 minutes during the test and after 5 minutes in the recovery phase with Lactase Scout lactate analyzer (EKF Diagnostics).

### 2.3 Randomized placebo‐controlled iron supplementation for 3 months

**Figure 1:**
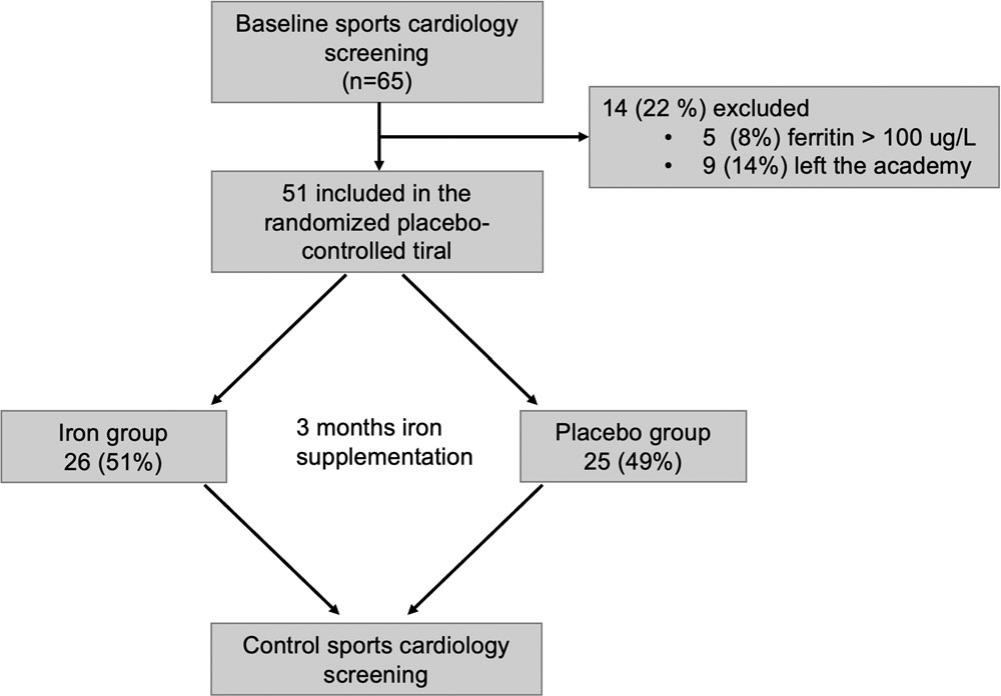
Placebo‐controlled trial design.

Before the study a desired effect size of 0.8 with a desired power (*β*) of 0.8 was selected for hypothesis testing. Using these parameters, the minimum number of samples required is 52 (26−26 in each group). Patient enrollment was performed by the physicians carrying out the detailed sports cardiology screening. Out of the 65 examined athletes, 51 were included in the randomized placebo‐controlled trial as they all had a ferritin level <100 μg/L, this number of sample size was sufficient to test our hypothesis. Therefore, in all the “U groups,” the subjects were equally randomized into iron supplementation (*n* = 26) and placebo (*n* = 25) groups. We used single blind, stratified randomization technique. All the athletes have been first assigned into age groups, then we generated the iron and the placebo groups by using simple randomization, with an equal allocation ratio, by referring to a table of random numbers. The randomization was performed by our statistician. Only the statistician and the dietitian (who assigned the iron or placebo tablets for the athletes) were unblinded during the study, physicians were blinded until the end of the study. In the iron group, we administered 3‐month oral iron supplementation as “Sorbifer durules 320 mg” ferrous sulfate tablets, which contains 100 mg elemental iron (II) and 60 mg ascorbic acid.^15^, ^18^ In the placebo group, we used 50 mg vitamin C. The dosage consisted of one tablet twice a day every second day to achieve better absorption and fewer side effects. After the 3‐month of treatment period, all the exams (patient history, resting ECG, laboratory tests, echocardiography, body composition analysis, CPET) were repeated (Figure 1).

### 2.4 Statistical analysis

Statistical analyses were performed using GraphPad Prism 8.0.1 program package. A Shapiro−Wilk's test was used to test the normality of the variables. Normal distribution data are presented as mean ± standard deviation, non‐normal distribution data are presented as median with interquartile range. Categorical variables are presented as percentages and frequencies. Comparisons of the means of continuous variables were performed using unpaired Welch's *t*‐tests instead of the traditional analysis of variance (ANOVA). ANOVA assumes that different groups have the same variance. To ensure that ANOVA could be used, a test for homoscedasticity would be required. However, it was shown that using such tests may inflate type 1 error rates and thus it is best to avoid them.^19^ Appropriate effect sizes for Welch's *t*‐test were computed with Hedges' *g*. The distributions of non‐normal continuous variables were compared by Mann−Whitney *U* tests. *p*‐value < .05 was considered to indicate statistical significance.

## 3 RESULTS

### 3.1 Study population

A detailed sports cardiology screening was applied in 65 male basketball players (age: 15.8 ± 1.7 years). All the athletes play in the same youth academy; thus, all of them train equally 20 h/week. Baseline characteristics, laboratory, echocardiography, and CPET results are shown according to age groups in the Supplement Table.

### 3.2 Findings of the sports cardiology screening

During the sports‐specific questionnaire no athlete reported any medical complaint, while positive family history was revealed in 9 (13.8%) cases.

According to the dietary consultation and the FFQ test, there were no vegetarians among the players. They consumed meat every day as well as food with a high iron content at least twice a week: 12% of them consumed fish, 64% whole grain products, 4% bran and sprouts, 27% dried legumes, 16% nuts, and 33% cocoa. All the athletes had diverse and balanced diet with adequate nutritional intake.

Due to high resting or exercise blood pressure values, 24‐hour ambulatory blood pressure monitoring exams were performed in 11 cases (17%). Hypertension was diagnosed in one case; angiotensin receptor blocker therapy was initiated.

Regarding the laboratory exams, no major alteration was found. In one case thalassemia minor was diagnosed based on laboratory parameters (mildly decreased hemoglobin [121 g/L], hematocrit [0.37], mean corpuscular volume [61.2 fL], and mean corpuscular hemoglobin [20 pg] with elevated red blood cell [6.06 T/L] and red blood cell distribution width [17.3%]). After hematology consultation, lifetime folic acid therapy was initiated.

Anemic iron deficiency (IDA) was not detected, but the frequency of IDNA was high (*n* = 60, 92%). No athlete had laboratory signs of infection. Hemoglobin and hematocrit levels did not show any difference between the age groups. The ferritin level increased with age. The difference in ferritin levels was significant between the U15 and U19 age groups (U15: 32.3 ± 24.6 vs. U19: 63.2 ± 22.9 μg/L, *p* < .005). Iron stores are least loaded in the youngest athletes (Supporting Information: Table).

On the resting ECG, the most common physiological ECG change was incomplete right bundle branch block (*n* = 42, 65%), which was present in more than half of the athletes. Sinus arrhythmia (*n* = 19, 29%) and sinus bradycardia (*n* = 14, 22%) were also regular among the athletes. T‐wave inversion in V1−V3 (*n* = 23, 35%) occurred more frequently in younger age groups (U15: 50% vs. U19: 19%, *p* < .05). Resting ECG examination revealed pathological T‐wave inversion in two players. To rule out structural heart disease, we performed cardiac MRI examination, which found no pathological changes.

On the echocardiography, we found only minor heart valve disease in 3 subjects (5%), which were mitral prolapse in two cases and mild pulmonary regurgitation in one case.

In one case, during the CPET exam two consecutive monomorphic six beats long wide QRS tachycardia were recorded in the restitution phase. The athlete was asymptomatic, and his resting ECG, echocardiography, and cardiac MRI was negative. According to the morphology, Belhassen tachycardia was established, and regular follow‐up was recommended. Impaired respiratory values were detected in one player during the CPET exam. The athlete was referred for a pulmonology examination where asthma was diagnosed, and therapy was initiated.

In the CPET exams, the maximum load showed a constant increase in the different age groups. The resting and peak HR had a declining trend; meanwhile HR recovery increased with age. The difference in VO_2_ max values was significant between the U15, U16, and U18, U19 age groups (U15 vs. U18, *p* = .002, U15 vs. U19, *p* = .001, U16 vs. U18, *p* = .009, U16 vs. U19, *p* = .003), the U19 group had the highest value. Maximum aerobic capacity, ventilation, and peak lactate increased with age. The average RER value in all age groups exceeded 1.10; they reached the maximum load. Altogether, the performance of the U19 group was the highest (Supporting Information: Table).

### 3.3 Randomized placebo‐controlled iron supplementation results

As a result of the 3‐month iron supplementation, 15% of the athletes reached the expected level of 100 μg/L ferritin values. In the iron group, the ferritin level significantly increased compared to the baseline assessment. The iron group had significantly higher ferritin levels compared to the placebo group, with the average increase of 37.5% ferritin. The correlation between VO_2_ max and ferritin levels could not be verified (*r* = .009, *p* = .95, *g* = 0.25) in this group of athletes. According to the CPET exam, there was no difference between the performance of the two groups. However, the aerobic capacity significantly improved in both groups. In the placebo group the blood iron level increased. However, we found no difference in any other parameter after the supplementation period (Table 1.).

**Table 1:** The effect of 3‐month iron supplementation.

	Baseline (B)				Supplementation (S)				*p* Value IG B versus S	*p* Value PG B versus S
	Iron group (IG) *n* = 26	Placebo group (PG) *n* = 25	*p*	Effect size (Hedge's *g*)	Iron group (IG) *n* = 26	Placebo group (PG) *n* = 25	*p*	Effect size (Hedge's *g*)		
Age (years)	15.6 ± 1.8	15.6 ± 1.7	.96	0.01	16.5 ± 1.8	16.2 ± 1.8	.66	0.12	.10	.23
Weight (kg)	70.8 ± 11.7	71.8 ± 14.7	.77	0.09	73.6 ± 10.6	74.8 ± 13.9	.72	0.1	.36	.46
Height (cm)	182.5 ± 6.3	182.0 ± 8.5	.83	0.06	184.4 ± 6.2	183.8 ± 7.3	.74	0.09	.26	.42
Sport experience (years)	7.0 ± 2.0	7.4 ± 2.3	.47	0.2	7.9 2.1	8.4 ± 2.3	.41	0.23	.10	.12
Body muscle mass (kg)	36.2 ± 5.2	36.5 ± 7.0	.86	0.05	37.0 ± 3.9	37.9 ± 6.4	.59	0.15	.53	.47
Body fat percent (%)	9.3 ± 3.7	10.0 ± 4.5	.60	0.14	9.4 ± 3.9	10.5 ± 4.5	.35	0.26	.93	.62
Laboratory results										
Hemoglobin (g/L)	152 ± 8.7	151 ± 12.3	.78	0.08	153 ± 7.9	153 ± 9.7	.96	0.01	.55	.50
Hematocrit	0.45 ± 0.02	0.44 ± 0.03	.45	0.21	0.45 ± 0.03	0.45 ± 0.02	.55	0.16	.59	.50
Blood iron (μmol/L)	18.2 ± 7.2	15.4 ± 6.7	.17	0.39	19.5 ± 6.4	19.7 ± 7.1	.90	0.03	.52	**.04**
TIBC (μmol/L)	73.5 ± 9.8	73.7 ± 12.8	.95	0.02	70.2 ± 6.7	72.3 ± 10.7	.41	0.02	.16	.68
sTfr (mg/L)	3.3 ± 0.8	3.6 ± 1.4	.37	0.27	3.6 ± 0.8	3.6 ± 0.9	.98	0.09	.19	.97
Transferrin (g/L)	2.9 ± 0.4	2.9 ± 0.5	.95	0.02	2.8 ± 0.3	2.9 ± 0.4	.44	0.29	.16	.63
Transferrin saturation (%)	25.5 ± 11.7	22.4 ± 12.0	.35	0.26	28.2 ± 10.9	29.1 ± 13.3	.79	0.1	.40	.08
Ferritin (μg/L)	45.5 ± 22.5	45.2 ± 28.5	.97	0.01	76.3 ± 35.1	54.7 ± 25.2	**.01**	**0.72**	**.0004**	.22
Cardiopulmonary exercise testing results										
Treadmill time (min)	12.0 ± 2.0	12.7 ± 2.2	.24	0.33	12.7 ± 1.9	13.5 ± 2.4	.21	0.35	.19	.24
Max load (Watt)	310 ± 48.5	327 ± 81.3	.38	0.25	336 ± 46.1	356 ± 77	.28	0.3	.05	.21
Resting HR (bpm)	71.7 ± 11.6	65.9 ± 16.6	.15	0.4	68.2 ± 10.4	69.2 ± 18.4	.81	0.07	.25	.51
Peak HR (bpm)	196.5 ± 9.8	195.9 ± 9.0	.83	0.06	195.2 ± 6.9	194.4 ± 9.0	.72	0.1	.59	.57
HR recovery	32.1 ± 8.4	32.7 ± 7.8	.80	0.07	27.9 ± 7.2	30.7 ± 9.5	.25	0.33	.06	.44
VO_2_ max (L/min)	3.7 ± 0.6	3.7 ± 0.8	.84	0.06	4.0 ± 0.5	4.2 ± 0.8	.37	0.25	**.02**	**.04**
VO_2_ max (mL/min/kg)	52.3 ± 4.3	52.3 ± 5.1	.98	0.01	55.0 ± 3.8	56.4 ± 5.6	.29	0.3	**.02**	**.01**
Peak lactate (mmol/L)	8.8 ± 2.5	9.1 ± 2.0	.67	0.12	9.1 ± 2.2	9.1 ± 2.6	.90	0.03	.65	.93

Abbreviations: B, baseline; HR, heart rate; IG, iron group; PG, placebo group; S, supplementation; sTfr, soluble transferrin receptor; TIBC, total iron binding capacity; VO_2_ max, maximal aerobic capacity.

## 4 DISCUSSION

In this study, we performed baseline and posttreatment evaluations on young basketball players focusing on their iron status and performance. We assessed the prevalence of ID at baseline according to age groups and found that IDNA is more prevalent in the younger age groups. We randomized IDNA athletes based on ferritin levels into iron and placebo groups. We administered oral iron supplementation for 3 months and found that this only affected ferritin levels but had no impact on sports performance.

During the sports cardiology screening, no major underlying cardiac disease was verified. In our study, IDNA was very frequent, affecting 92% of our young basketball players. In parallel with our study, Shoemaker et al. found that ID rates in young athletes between the ages of 8 and 16, were similarly high. In their study, 65% of the boys and 86% of the girls had low ferritin levels, even though they used a much lower ferritin cut‐off value (30 μg/L).^20^ As iron has a key role in oxygen transport and energy production, it is essential for optimal sports performance. Therefore, even IDNA can impair sports performance by negatively affecting iron‐dependent oxidative enzymes and respiratory proteins. ID is more common among athletes since exercise related increased iron demand, iron loss, and blockage of iron absorption due to hepcidin bursts propagate the reduction of iron storage.^21^, ^22^, ^23^

Most of the previous studies investigate the iron status of endurance athletes. However, this may be more important in tactical sports, where skills, speed, and decision making under fatigue, and stress are more prominent. Dubnov et al. examined the iron status of top‐level male and female basketball players (*n* = 108).^24^ IDNA was found in 22% of the athletes; however, they used 20 μg/L cut‐off value. Anemia was verified in 25% of the athletes. These results support our findings; even though they used a lower cut‐off value, more than 50% of the athletes were affected with ID. Furthermore, the findings reinforce the need to monitor the iron status not only for endurance athletes. According to the nature of basketball, the leading cause of exercise induced iron loss could be the red blood cell destruction caused by mechanical stress. However, exercise induced hemolysis can be detected in several sports modalities; the most severe form may be observed after running.^25^, ^26^, ^27^, ^28^ In basketball, contact injuries resulting in hematoma may also contribute to iron loss. Heavy sweating and skin desquamation are also mechanisms which can impact the iron status negatively.^29^

In young athletes, the rapid growth and development combined with increased physical activity predisposes children to develop ID. Due to the growing blood volume and muscle mass, they have an extra iron demand; hence identifying ID in this population is essential.^30^ In our study, we found no IDA athletes, all of them had normal blood count values.

In athletes, there is no widely accepted cut‐off value for ferritin and there is no guideline for iron supplementation dose and length. A meta‐analysis which included 17 studies found that low ferritin levels can be significantly increased with iron treatment. They mainly used the ferritin cut‐off <20 μg/L with a supplementation period of an average of 61 days. Most studies examined only female athletes.^15^ In our study, we decided to choose a higher ferritin cut‐off, as this controlled monitoring can signal and prevent the reduction of iron stores in these young developing male group of athletes. As ID is a frequently seen comorbidity in heart failure patients, this higher ferritin cut‐off (≤100 μg/L) was implemented in the heart failure guideline in 2016 and has been used to decrease symptoms, improve functional capacity, and the quality of life.^11^, ^12^, ^13^, ^31^ ID has been proved to worsen the symptoms and the prognosis, regardless of the presence of anemia. Although, athletes, especially the young athletes, have a high iron demand, the optimal cut‐off for iron supplementation may not be as high as in heart failure patients.

In our study, after the 3‐month supplementation the ferritin level was significantly higher in the iron group than in the placebo group, but only a small portion of the athletes (15%) reached our expected ferritin level. Several studies assessed the effect of oral iron supplementation on iron status laboratory parameters in athletes with IDNA. A meta‐analysis by DellaValle et al. has concluded that 6−8 weeks of at least 100 mg of iron‐sulfate supplementation improves the level of ferritin or prevents the further decline of ferritin level in female athletes.^32^ In a study, Villanueva et al. assessed the effect of oral iron supplementation on laboratory parameters in elite soccer players, where they also found a significant increase in ferritin levels after 3 weeks of supplementation.^33^ Parallel to our results, Kang et al. did not find a blood count increase in IDNA athletes after iron supplementation.^34^ According to these results iron supplementation in IDNA athletes only refills the iron storages without any change in blood count parameters. This may have an indirect impact on the oxygen transport with modifying the iron‐dependent oxidative enzymes and respiratory proteins. We found statistically significant increase in blood iron concentration in the placebo group. However, we did not consider that clinically relevant, since blood iron levels can be influenced by a number of other factors: diurnal variation, fasting status, inflammatory status. A study examined the role of the serum iron level in the diagnosis of children (<18 years) with ID. They concluded that the serum iron level is no longer recommended by guidelines and that it should not be used to establish the iron status.^35^

In our study, the maximal aerobic capacity increased in both the iron and the placebo groups without any difference after the supplementation between the iron and the placebo groups. This finding can be explained by the athletes' improved performance during the season.

According to iron supplementation protocols, 6−12 weeks of oral iron supplementation is required to replenish the iron stores.^3^ In our study, we administered iron for 3 months and we found no difference in performance between the iron and the placebo groups. In the literature, it is also questionable whether iron supplementation improves athletic performance in IDNA athletes. In a meta‐analysis by Rubero et al., 12 studies were selected with a total of 283 participants to assess the impact of iron supplementation on performance.^10^ Performance improvement was detected in six studies in which ferritin cut‐off was ≤20 μg/L. However, there was only one study which continued the iron supplementation for 3 months, the mean supplementation period in the others was only 6−8 weeks. In contrast to our study, we used a longer period of oral supplementation with a higher ferritin cut‐off, but no performance increase was detectable.

In our study, there were no side effects of the iron supplementation. A study by McCormick et al. has examined the daily or alternate day oral iron supplementation effectiveness in runners.^36^ They also concluded that the alternate day supplementation was effective and had lower gastrointestinal side effects. Regarding our results, the applied dosage and the frequency of the supplementation were adequate to prevent the gastrointestinal side effects.

## 5 CONCLUSIONS

Sports cardiology screening of young athletes is essential to discover not only life‐threatening abnormalities but also performance limiting factors, such as ID. In young athletes, ID is common and more pronounced in the youngest age groups; therefore, the early screening of ID is essential. In our randomized placebo‐controlled trial, after the 3‐month oral iron supplementation the ferritin levels increased in the iron group without significant performance improvement between the placebo and the iron group; however, only a small portion of the athletes achieved the expected cut‐off value. Our study highlights the need of age‐corrected ferritin cut‐off value or using a combined interpretation of the iron panel in young athletes. Further studies are needed to establish the optimal iron supplementation form, period, and dose in athletes.

## AUTHOR CONTRIBUTIONS

Study design and conceptualization, methodology was performed by Emese Csulak, Titanilla Takács, Brigitta Babis, Laura Horváth, Petra Márton, Nóra Sydó, and Béla Merkely. Statistical analysis and the data curation was performed by Levente Staub, Emese Csulak, Brigitta Babis, Titanilla Takács, and Nóra Sydó. Emese Csulak, Titanilla Takács, Brigitta Babis, Petra Márton, Laura Horváth, Bálint Lakatos, Attila Kovács, Zsófia Dohy, Hajnalka Vágó, Levente Staub, Liliána Erzsébet Szabó, Nóra Sydó, and Béla Merkely wrote the original draft of the paper. Emese Csulak, Titanilla Takács, Brigitta Babis, Laura Horváth, Petra Márton, Bálint Lakatos, Attila Kovács, Zsófia Dohy, Levente Staub, Liliána Erzsébet Szabó, Hajnalka Vágó, Nóra Sydó, and Béla Merkely contributed to reviewing and editing. Nóra Sydó and Béla Merkely did the supervision. All authors have read and agreed to the published version of the manuscript.

## CONFLICT OF INTEREST STATEMENT

The authors declare no conflict of interest.

## Supporting information

Supporting information.

## Data Availability

The data set presented in this study are available on request from the corresponding author. Due to patient's data, privacy data are not made available publicly.
