# Supplementary material for: Iron deficiency in young basketball players: Is a 100 μg/L ferritin cut‐off appropriate for iron supplementation?: Results of a randomized placebo‐controlled study
Source: Clin Cardiol. 2023 Sep 19;46(9):1116–23. doi: 10.1002/clc.24117 (PMC10540028; doi:10.1002/clc.24117)
Supplement: Supplementary file 1 — Supporting information. [file CLC-46-1116-s001.docx]

**Supplement Table.** Results of the sports cardiology screening according to age.

|  | **U15**  **(n=14; 22%)** | **U16**  **(n=17; 26%)** | **U18**  **(n=18; 28%)** | **U19**  **(n=16; 25%)** | |
| --- | --- | --- | --- | --- | --- |
| **ECG results** | | | | | |
| Sinus bradycardia | 0 (0%) | 3 (18%) | 3 (17%) | 8 (50%) | |
| Sinus arrhythmia | 6 (43%) | 3 (18%) | 4 (22%) | 6 (38%) | |
| Incomplete RBB | 8 (57%) | 12 (71%) | 12 (67%) | 10 (63%) | |
| LV hypertrophy | 1 (7%) | 2 (12%) | 2 (11%) | 2 (13%) | |
| T-wave inversion in V1-V3 | 7 (50%) | 7 (41%) | 8 (44%) | 3 (19%) | |
| **Laboratory results** | | | | | |
| Hemoglobin [135-170] (g/L) | 151 ± 10.7^1^ | 153 ± 9.7^1^ | 155.5 ± 9.8^1^ | 148.6 ± 11.5^1^ | |
| Hematocrit [0.39-0.52] | 0.4 ± 0.0^1^ | 0.5 ± 0.0^1^ | 0.5 ± 0.0^1^ | 0.4 ± 0.0^1^ | |
| MCV [80-99] (fL) | 84.9 ± 2.3^1^ | 86.6 ± 3.5^1^ | 84.7 ± 2.9^1^ | 84.1 ± 7.3^1^ | |
| MCH [27-34] (pg) | 28.7 ± 1.2^1^ | 29.5 ± 1.4^1^ | 29.2 ± 1.3^1^ | 28.6 ± 2.8^1^ | |
| RDW [11.5-15.0] (%) | 12.6 ± 1.1^1^ | 12.2 ± 0.5^1^ | 12.0 ± 0.7^1^ | 12.6 ± 1.5^1^ | |
| Blood iron [12.5-32.2] (μmol/L) | 13.9 ± 8.0^1^ | 16.9 ± 6.8^1^ | 18.0 ± 5.3^1^ | 19.0 ± 6.5^1^ | |
| TIBC [45-81] (μmol/L) | 81.3 ± 8.2^2^ | 77.2 ± 9.3^2^ | 69.0 ± 8.0^1^ | 65.8 ± 11.1^1^ | |
| sTrf [2.2-5.0] (mg/L) | 4.1 ± 1.4^2^ | 3.3 ± 0.8^1,2^ | 3.0 ± 0.8^1^ | 3.2 ± 0.8^1,2^ | |
| Transferrin [2.0-3.6] (g/L) | 3.2 ± 0.3^2^ | 3.1 ± 0.4^2^ | 2.8 ± 0.3^1^ | 2.6 ± 0.4^1^ | |
| Transferrin saturation [16-45] (%) | 17.2 ± 9.6^2^ | 22.6 ± 10.2^1,2^ | 26.3 ± 7.6^1^ | 30.7 ± 14.0^1^ | |
| Ferritin [6-320] (μg/L) | 32.3 ± 24.6^1^ | 43.2 ± 22.5^1,2^ | 45.2 ± 19.3^1,2^ | 63.2 ± 22.9^2^ | |
| Ferritin < 50 μg/L (n, %) | 13 (93 %)^3^ | 8 (47 %)^2^ | 10 (56 %)^2^ | 3 (19 %)^1^ | |
| Ferritin < 20 μg/L (n, %) | 5 (36 %)^3^ | 3 (18 %)^2^ | 2 (11 %)^1^ | 1 (6 %)^1^ | |
| Vitamin-D [30-60] (μg/L) | 31,5±4,9^1^ | 33,6±14,7^1,2^ | 39,3±13,2^2^ | 35,9±10,5^1,2^ | |
| Creatin-kinase [3-190] (U/L) | 246,1±213,3^1^ | 558,1±386,1^2^ | 484±399,1^2^ | 859,3±726,9^2^ | |
| **Echocardiography results** | | | | | |
| Ejection fraction (%) | 62,9±3,3^1^ | 62,6±3,0^1^ | 63,3±2,3^1^ | 64,1±2,1^1^ | |
| Septal wall thickness (mm) | 9,1±1,7^2^ | 7,8±1,4^1^ | 10,1±0,9^2^ | 9,5±0,8^2^ | |
| Posterior wall thickness (mm) | 8,4±0,8^1^ | 8,2±1,4^1^ | 9,8±0,8^2^ | 9,0±1,0^1^ | |
| LV end-diastolic diameter (mm) | 47,6±4,6^1^ | 47,5±5,1^1^ | 48,3±4,0^1^ | 50,4±2,9^1^ | |
| RV diameter (mm) | 34,3±2,8^1^ | 34,5±3,6^1,2^ | 36,2±3,0^1,2^ | 36,6±2,1^2^ | |
| **Cardiopulmonary exercise testing results** | | | | |  |
| Treadmill time (min) | 11.5±1.5^1^ | 13.1±1.6^2^ | 12.2±2.2^1,2^ | 12.7±2.6^1,2^ | |
| Max load (Watt) | 258.2±52.9^1^ | 313.6±32.1^2^ | 352.7±62.7^2,3^ | 360.6±52.2^3^ | |
| Resting HR (bpm) | 81.5±7.8^3^ | 62.5±15.4^1,2^ | 70.0±12.3^2^ | 59.9±8.9^1^ | |
| Peak HR (bpm) | 197.0±7.6^1^ | 196.5±9.8^1^ | 197.4±9.1^1^ | 193.8±9.9^1^ | |
| HR recovery (1/min) | 30.4±4.9^1^ | 31.5±7.9^1^ | 31.6±5.7^1^ | 36.3±10.7^1^ | |
| VO_2_ max (L/min) | 3.2±0.6^1^ | 3.5±0.4^1^ | 4.0±0.5^2^ | 4.2±0.6^2^ | |
| VO_2_ max (mL/min/kg) | 53.1±4.2^1^ | 51.4±3.2^1^ | 51.1±5.2^1^ | 53.5±5.5^1^ | |
| O_2_ pulse (mL/bpm) | 17.4±4.8^1^ | 18.4±2.1^1^ | 20.7±2.7^1,2^ | 22.8±3.7^2^ | |
| VE (L/min) | 119.5±23.3^1^ | 137.4±16.4^2^ | 147.9±19.5^2^ | 150.3±30.0^2^ | |
| VE/VCO2 | 31.5±2.5^1,2^ | 33.1±3.9^2^ | 30.3±3.2^1,2^ | 29.7±2.7^1^ | |
| RER | 1.1±0.1^1^ | 1.2±0.1^1^ | 1.2±0.1^1^ | 1.2±0.1^1^ | |
| Peak lactate (mmol/L) | 8.3±1.2^1^ | 9.1±2.1^1^ | 8.9±2.6^1^ | 9.7±2.8^1^ | |

Abbreviations: U: under age group, LV: left ventricle, RBB: right bundle branch block, MCV: mean corpuscular volume, MCH: mean corpuscular hemoglobin, RDW: red blood cell distribution width, TIBC: total iron binding capacity, sTfr: soluble transferrin receptor, LV: left ventricle, RV: right ventricle, HR: heart rate, VE: ventilation, RER: respiratory exchange ratio, VO_2_ max: maximal aerobic capacity

Laboratory reference values are presented in brackets after all parameters. Superscripts represent differences among the groups significant at P <0.05.
